# Genetic architecture and major genes for tuber skin texture in potato

**DOI:** 10.1093/hr/uhag102

**Published:** 2026-03-13

**Authors:** Renhong Zhang, Shuo Wang, Jiangang Liu, Yinqiao Jian, Junhong Qin, Liping Jin, Ming He, Jianfei Xu

**Affiliations:** State Key Laboratory of Vegetable Biobreeding, Key Laboratory of Biology and Genetic Improvement of Tuber and Root Crop of Ministry of Agriculture and Rural Affairs, Institute of Vegetables and Flowers, Chinese Academy of Agricultural Sciences, Beijing 100081, China; State Key Laboratory of Vegetable Biobreeding, Key Laboratory of Biology and Genetic Improvement of Tuber and Root Crop of Ministry of Agriculture and Rural Affairs, Institute of Vegetables and Flowers, Chinese Academy of Agricultural Sciences, Beijing 100081, China; State Key Laboratory of Vegetable Biobreeding, Key Laboratory of Biology and Genetic Improvement of Tuber and Root Crop of Ministry of Agriculture and Rural Affairs, Institute of Vegetables and Flowers, Chinese Academy of Agricultural Sciences, Beijing 100081, China; State Key Laboratory of Vegetable Biobreeding, Key Laboratory of Biology and Genetic Improvement of Tuber and Root Crop of Ministry of Agriculture and Rural Affairs, Institute of Vegetables and Flowers, Chinese Academy of Agricultural Sciences, Beijing 100081, China; State Key Laboratory of Vegetable Biobreeding, Key Laboratory of Biology and Genetic Improvement of Tuber and Root Crop of Ministry of Agriculture and Rural Affairs, Institute of Vegetables and Flowers, Chinese Academy of Agricultural Sciences, Beijing 100081, China; State Key Laboratory of Vegetable Biobreeding, Key Laboratory of Biology and Genetic Improvement of Tuber and Root Crop of Ministry of Agriculture and Rural Affairs, Institute of Vegetables and Flowers, Chinese Academy of Agricultural Sciences, Beijing 100081, China

## Abstract

Potato (*Solanum tuberosum* L.) is a globally important tuber crop and a vital component of the food system. Tuber skin texture is a key quality trait that influences market appearance and is closely associated with resistance to biotic and abiotic stresses as well as tolerance to mechanical damage. However, the genetic basis and regulatory mechanisms underlying this trait remain poorly understood. In this study, we investigated the genetic and molecular mechanisms underlying potato tuber skin texture. A quantitative trait locus (QTL) for tuber skin texture was mapped to a 1.94-Mb interval on chromosome 4 using bulked segregant analysis of a segregating population derived from a cross between russet-skinned variety Innovator and smooth-skinned variety Zhongshuzao43 (Z43). The tuber skin of Innovator contained more cell layers than Z43 and developed progressive cracking during tuber expansion. Innovator also exhibited lower suberin content but higher lignin accumulation in tuber skin compared to Z43. Transcriptome profiling across multiple developmental stages identified a distinct gene expression cluster enriched in pathways related to lignin and suberin biosynthesis. Integrating genes within the QTL with this expression cluster revealed *StPXG4*, which encodes a peroxygenase, as strongly correlated with skin texture. *StPXG4* showed significantly higher expression in commercial smooth-skinned varieties than in russet-skinned varieties. Co-expression analysis further identified two potential upstream regulators of *StPXG4*, namely *StMYB103* and *StMYB58*. These findings provide key insights into the genetic regulation of tuber skin texture and identify candidate genes that could be targeted to improve tuber appearance and stress tolerance through molecular breeding.

## Introduction

Potato (*Solanum tuberosum* L.), the world’s third-largest food crop after rice and wheat, plays a crucial role in global food security due to its nutritional value and stable yields [1, 2]. The tuber skin, as the outermost protective layer, is essential for minimizing water loss, resisting biotic and abiotic stresses, and providing mechanical strength [3]. Moreover, the texture of tuber skin is an important appearance quality trait of potato tubers [4]. Although smooth-skinned tubers are preferred for their attractive appearance and strong consumer acceptance, they are more susceptible to bruising than russet tubers. In contrast, russet tubers develop a thicker periderm that confers improved resistance to soil-borne diseases and greater tolerance to mechanical damage compared with smooth-skinned tubers [5–8].

The texture of tuber skin is influenced by the interactions between genetic and environmental factors—such as temperature, humidity, and nutrient availability—and reflects the coordinated processes of cell growth, division, differentiation, and metabolism [9–11]. Earlier studies suggested that tuber skin texture is controlled by complementary gene interactions, based on segregation ratios observed in progeny from crossing experiments [12]. De Jong later supported this model and proposed a more refined hypothesis involving three independently segregating dominant genes, all of which must be present for the russet phenotype to appear [13]. To date, multiple genetic intervals associated with potato skin texture have been tentatively mapped to 11 chromosomes [14–16]. However, the key genetic intervals and underlying genetic basis remain unclear.

Russet skin is characterized by the tight adherence of ‘old’ phellem fragments to newly formed phellem layers [9]. Phellem, phellogen, and phelloderm are the major components of the periderm of the potato tuber [17]. Throughout tuber development, the phellogen remains active and generates new layers of phellem (skin) cells [18]. In smooth-skinned varieties, the outer suberized cell layers regularly slough off, maintaining a thin and glossy skin surface [19]. Conversely, in russet varieties, old phellem cells do not detach but instead accumulate. As the tuber expands, this accumulation leads to cracking and results in a thick and rough skin [20].

The phellem is composed of various cell wall components, including suberin, lignin, cellulose, and hemicellulose, that collectively form a robust barrier essential for protection and physiological regulation [21, 22]. Notably, significant differences in cell wall composition have been reported between smooth and russet skins [23]. For example, the cultivar ‘May Queen’ is predisposed to russeting under field conditions due to its inherently thicker periderm, higher pectin, and cellulose content [19].

Ferulic acid, α,ω-dicarboxylic acids, and ω-hydroxy acids constitute the main components of potato suberin [24]. Silencing the suberin feruloyl transferase (*StFHT*) reduces the levels of ferulic acid esters and suberin, while the periderm becomes thicker and rougher, and the tuber exhibits a rough-skin-like phenotype [25]. Recent studies show that the transcription factors (TFs) *StMYB24* and *StMYB144* positively regulate the *StFHT* expression, thereby promoting the accumulation of suberin monomers [26]. Additionally, the downregulation of *StSnakin-2* has been linked to increased lignin biosynthesis and a higher incidence of skin cracking [27]. However, the genetic basis, key regulatory factors, and mechanisms underlying potato tuber skin texture remain largely unclear.

We observed that the tuber skin of the russet variety, Innovator, gradually cracked and accumulated on the surface during tuber development. In contrast, the tuber skin of the smooth variety, Z43, was tightly packed and well organized. Compared with Z43, Innovator exhibited lower suberin content but higher lignin content in the tuber skin. Through bulked segregant analysis (BSA), a quantitative trait locus (QTL) associated with tuber skin texture was mapped to chromosome 4, within the 1.86–3.80-Mb interval. By integrating QTL mapping with transcriptome profiling, *StPXG4* was identified as a critical candidate gene regulating tuber skin texture, along with potential upstream regulators, *StMYB103* and *StMYB58*. This study revealed significant genetic regions underlying potato tuber skin texture and identified critical candidate genes within these loci.

## Results

### Tuber skin texture is related to the structure and organization of skin layers

**Figure 1:**
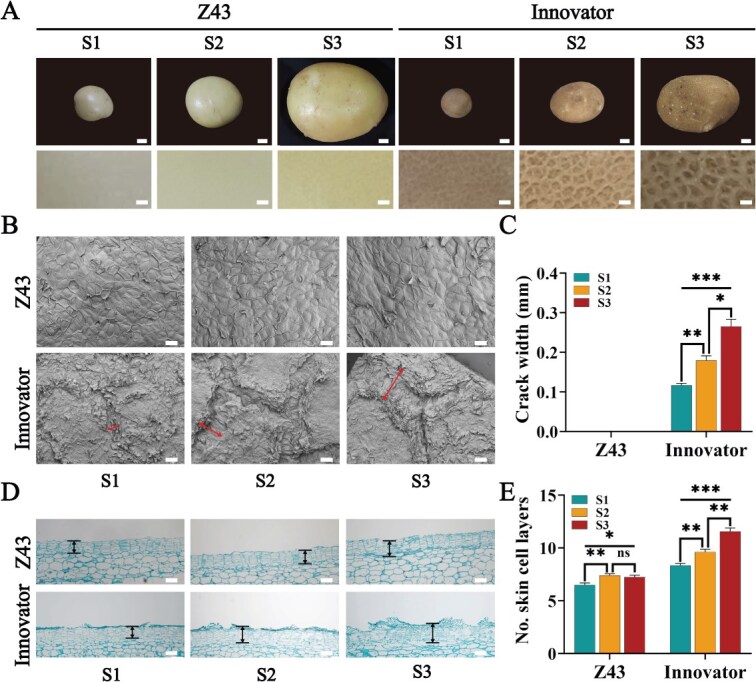
The tuber skin structure and cellular arrangement differ between Innovator and Z43. (A) Surface appearance of tuber skin in Z43 and Innovator at tuber diameters of 3, 6, and 10 cm (abbreviated as S1, S2, and S3, respectively). Scale bar = 1 cm (upper panel) and 1 mm (lower panel). (B) SEM images of tuber skins at stages of S1, S2, and S3. Arrows indicate the cracks of the tuber skin. Scale bar = 0.1 mm. (C) Crack width in the skin of Z43 and Innovator. Data are presented as mean ± SE (*n* = 18). All differences were statistically significant by two-way ANOVA and Tukey’s multiple-comparison test: ^*^P < 0.05; ^**^P < 0.01; ^***^P < 0.001. (D) Images of tuber skin layer arrangement. The lines demarcate the area for the quantification of skin cell layers. In the Innovator variety, the quantified layers correspond to the area of retained, fissured phellem. Scale bar = 100 μm. (E) Tuber skin cell layers of Z43 and Innovator. Data are presented as mean ± SE (*n* = 9). All differences were statistically significant by two-way ANOVA and Tukey’s multiple-comparison test: ^*^P < 0.05; ^**^P < 0.01; ^***^P < 0.001; ns, not statistically significant.

Smooth-skinned potato varieties are characterized by a clean, shiny appearance, in contrast to the darker hue and rough skin of russet potatoes [28]. To investigate the developmental mechanisms underlying skin texture, we selected a smooth-skinned variety, Z43, and a typical russet-skinned variety, Innovator [29], for comparative analysis. Tubers were sampled at ~3, 6, and 10 cm in diameter during development (hereafter referred to as S1, S2, and S3, respectively) (Fig. 1). The tuber skin of Z43 remained consistently smooth across all three developmental stages (Fig. 1A). In contrast, the skin of Innovator gradually developed a visually reticulated pattern as tubers increased in size (Fig. 1A).

To further examine structural differences, scanning electron microscopy (SEM) was used to observe the tuber skins. The skin of Z43 tubers was densely packed and tightly arranged, resulting in a uniform and compact surface. In contrast, the skin of Innovator tubers displayed distinct cracks that became progressively wider with tuber growth (Fig. 1B and C), contributing to its characteristic rough texture.

To gain clearer insight into the internal skin structure of both varieties, paraffin sectioning was performed. Throughout the tuber expansion process, the skin layer cells of Z43 remained tightly and orderly arranged (Fig. 1D). In contrast, Innovator tubers exhibited evident skin fragmentation beginning at the S1 stage, with increasing severity at S2 and S3 (Fig. 1D). Furthermore, the number of skin cell layers of Innovator tubers increased significantly in parallel with the severity of cracking (Fig. 1E). These findings indicate that the structural differences between smooth and russet skins are closely associated with the organization and proliferation dynamics of skin layer cells.

### The suberin and lignin contents within the tuber skins differ significantly between smooth-skinned and russet-skinned tubers

**Figure 2:**
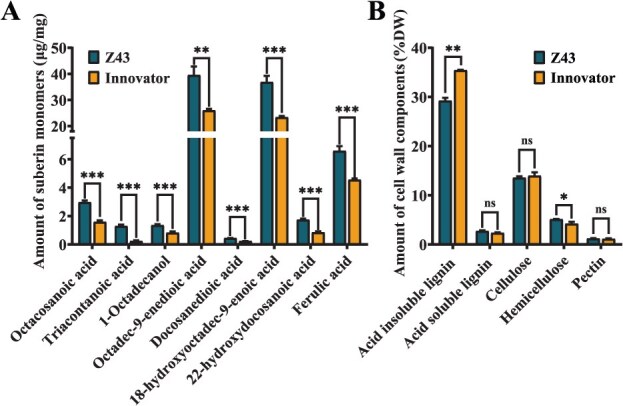
The contents of suberin and lignin vary in the tuber skins of Innovator and Z43. Suberin monomer content (A) and cell wall component content (B) in tuber skin of Innovator and Z43. Data are presented as mean ± SE. ^*^P < 0.05, ^**^P < 0.01, ^***^P < 0.001, Student’s *t*-test.

Suberin is an important component of the tuber skin [17]. We measured the suberin contents in the tuber skin of Z43 and Innovator, identifying eight monomeric compounds that were significantly more abundant in Z43 than in Innovator (Fig. 2A). Among these, two major chromatographic peaks corresponded to 18-hydroxy-9-octadecenoic acid and octadec-9-enedioic acid, members of the ω-hydroxyacids and α,ω-diacids, respectively (Fig. 2A; Fig. S1), both of which have previously been identified as primary suberin monomers [30]. Overall, the content of suberin was significantly higher in the tuber skin of Z43 compared with Innovator (Fig. 2A). It has been reported that genes involved in the biosynthesis of cell wall components are significantly upregulated in russet skins [27]. Therefore, cell wall composition was further examined, and it was found that the acid-insoluble lignin content in the tuber skin of Innovator was significantly higher than that in Z43 (Fig. 2B).

To further explore the association between suberin and lignin accumulation and skin texture, two russet- and two smooth-skinned progeny lines were randomly selected from the F_1_ population for quantification of suberin and lignin contents. Consistent with the detection results in Innovator and Z43, the smooth-skinned progeny lines exhibited significantly higher suberin accumulation, yet markedly lower lignin content, than those in russet-skinned progenies (Fig. S2). These results demonstrate that suberin and lignin levels are tightly related to periderm texture.

### The genetic region associated with tuber skin texture is located on chromosome 4

To investigate the genetic basis of tuber skin texture, an F₁ population was generated by crossing Z43 (♀) and Innovator (♂), resulting in 272 progeny individuals. Tuber skin texture was evaluated over two consecutive years (2023 and 2024) in Hebei and Inner Mongolia, respectively. The trait was visually scored on a scale of 1 (smooth) to 5 (russet) according to the National Standard for DUS Testing of Potato Varieties (GB/T 19557.28) (Table S1). The distribution of tuber skin texture in the F₁ population approximated a normal distribution, with highly consistent patterns across both years and locations (*r* ≥ 0.75) (Fig. S3), indicating stable phenotypic expression. Broad-sense heritability (*H*^2^) was estimated at 93.1% based on phenotypic data collected across two years and two locations, a value highly consistent with previous reports [14, 31].

To identify QTL associated with tuber skin texture, BSA was performed. Following resequencing and quality filtering, high-quality clean data were obtained: Z43 had 51.78 Gb (99.09% coverage), Innovator had 56.4 Gb (99.13% coverage), the smooth pool had 123.63 Gb (98.59% coverage), and the russet pool had 131.50 Gb (95.06% coverage) (Table S2). After filtering, a total of 28 482 127 single nucleotide polymorphisms (SNPs) and 4 533 311 small indels (1–50 bp) were identified. Among the SNPs, 692 868 were synonymous and 833 293 were nonsynonymous. The majority of SNPs were located in intergenic regions (Fig. S4; Table S3), and these variants were evenly distributed across all 12 chromosomes (Fig. S5).

**Figure 3:**
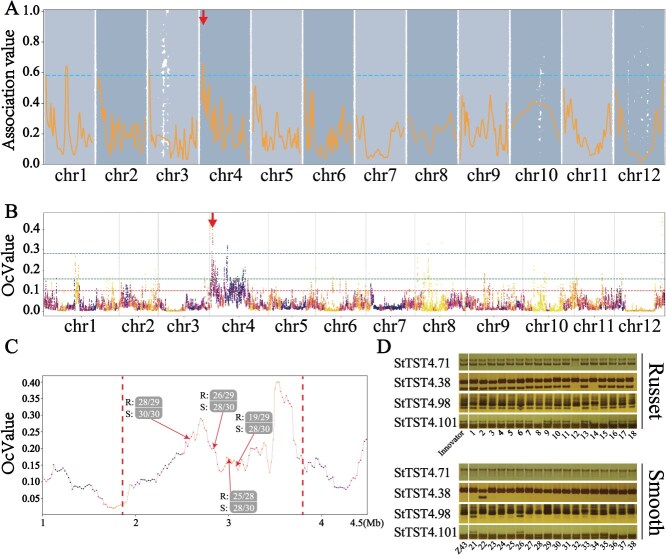
QTL mapping for the tuber skin texture. (A) Scatter plot of QTL mapping using DeepBSA. The *y*-axis shows the association value of SNPs calculated by the deep learning algorithm. The solid line represents the LOESS fit across all data points. The dashed horizontal line indicates the significance threshold, defined as 3 SD above the genome-wide median. The arrow marks the tuber skin texture QTL interval. (B) QTL mapping using OcBSA. The *y*-axis displays the OcValue, a metric quantifying the difference in allele frequency between the two constructed OcPools. The uppermost horizontal line denotes the top 0.1% of all OcValues. (C) The distribution of indel markers within the QTL. The box represents the number of pooled individuals in which the genotype of the indel markers matched the corresponding potato skin texture phenotype. R and S represent russet skin and smooth skin, respectively. The dashed line highlights the range of the QTL. (D) Validation of four indel markers in the F_1_ population. The genotypes of parents and a portion of extreme progeny are shown.

To map QTL associated with tuber skin texture, we employed two BSA-based methods—DeepBSA and OcBSA. Genomic intervals consistently identified by both approaches were prioritized as high-confidence regions for subsequent validation and functional characterization. Both approaches detected a significant peak on Chromosome 4, with overlapping intervals: DeepBSA mapped the region to 1.86–3.17 Mb, while OcBSA identified 2.3–3.8 Mb (Fig. 3A and B; Table S4). In addition, *k*-mer analysis revealed a marked enrichment of russet bulk-specific *k*-mers in the 2.0–3.8-Mb interval on the same chromosome (Fig. S6). To encompass the full potential of the QTL region, we selected the broader interval of 1.86–3.80 Mb for further analysis.

To validate this QTL region, four significant indel markers (*P* < 0.05) were developed, evenly spaced between 2.56 and 3.7 Mb (Fig. 3C and D; Table S5). Genotyping results revealed that the average concordance rate of these four markers in the F₁ population exceeded 80%, with three markers showing concordance rates above 90% (Table S6), confirming the reliability of the identified locus. These findings collectively indicate that a major QTL on Chromosome 4 is strongly associated with tuber skin texture.

### Transcriptome analysis screened candidate genes associated with tuber skin texture

**Figure 4:**
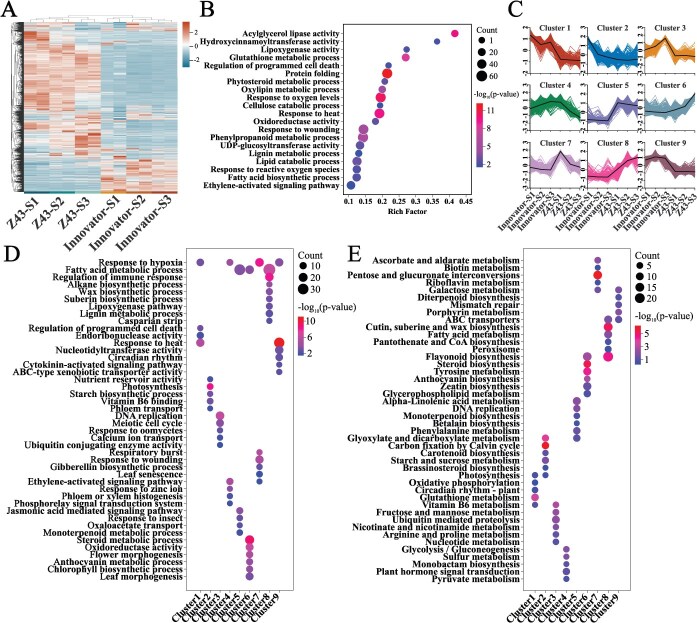
Transcriptome analysis of the tuber skin at different developmental stages. (A) Hierarchical cluster analysis of DEGs. In the heatmaps, columns represent samples and rows represent genes. (B) GO enrichment analysis of DEGs. (C) *K*-means clustering analysis reveals nine clusters of DEGs with distinct expression patterns. GO (D) and KEGG (E) term enrichment analysis provides insights into the functional significance of the nine clusters. The color intensity and size of each dot represent the −log_10_ (*P*-value) and the number of genes, respectively.

We conducted RNA sequencing of tuber skins from Z43 and Innovator at three developmental stages (S1, S2, and S3). Following quality control, a total of 539 342 254 clean reads were obtained, with ~84.58% successfully mapped to the reference genome. The dataset exhibited high-quality metrics, including a GC content of 42.23% and a Q30 score of 93.53% (Table S7). Principal component analysis (PCA) of the differentially expressed genes (DEGs) revealed distinct transcriptomic profiles between the two genotypes. PC1, accounting for 58.52% of the total variance, distinctly separated Z43 from Innovator, with all Innovator samples clustering consistently along this component. PC2, which explained 10.76% of the variance, further separated the Z43 tuber skin samples across the three developmental time points (S1, S2, and S3) (Fig. S7). This pattern was further confirmed by hierarchical clustering analysis (Fig. 4A), supporting the reliability of the RNA sequencing data. The DEGs were significantly enriched in lipoxygenase activity, fatty acid biosynthesis, and cellular metabolic pathways involved in cellulose and lignin metabolism (Fig. 4B).

To further explore functional differences between smooth- and russet-skinned tubers, we applied a K-means clustering algorithm to divide the DEGs into nine clusters, each characterized by a unique expression profile (Fig. 4C). Gene Ontology (GO) enrichment analysis revealed that the genes in cluster eight were significantly enriched in biological processes associated with cell wall biogenesis and suberin biosynthesis (Fig. 4D). Furthermore, the expression levels of these genes were markedly higher in Z43 compared with Innovator (Fig. 4C). Notably, the Kyoto Encyclopedia of Genes and Genomes (KEGG) pathways for cutin, suberin, and wax biosynthesis were significantly enriched in cluster eight (Fig. 4E). These findings suggest that cluster eight may contain key regulators involved in tuber skin texture.

### Identification of candidate genes involved in regulating tuber skin texture

**Figure 5:**
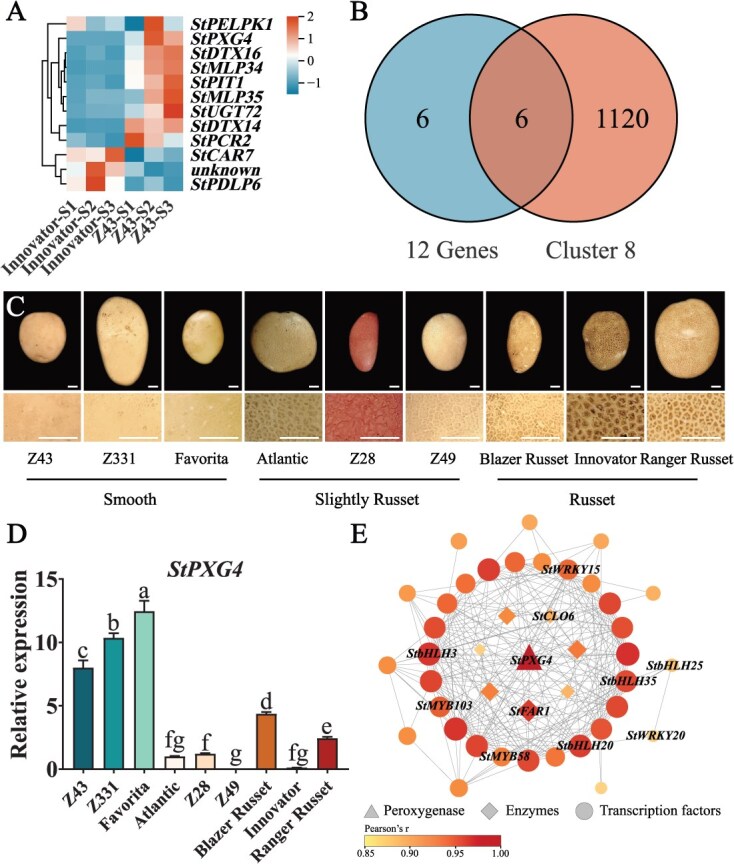
Identification of key candidate genes associated with tuber skin texture. (A) Heatmap of candidate gene expression. (B) Venn diagram illustrates the overlapping DEGs between cluster eight and the candidate genes. (C) Surface appearance of nine commercial varieties exhibiting varying degrees of skin texture. Scale bars = 1 cm. (D) Expression of *StPXG4* in nine commercial varieties. Data are presented as mean ± SE (*n* = 3). Lowercase letters indicate statistically significant differences at *P* < 0.05 (one-way ANOVA and Tukey’s multiple-comparison test). (E) Correlation network of *StPXG4* with the TFs and suberin biosynthesis-related genes in cluster eight. The sizes and colors of the circles are positively correlated with the strength of correlation.

To identify key candidate regulators of tuber skin texture, we focused on DEGs located within the previously identified QTL region. Among a total of 161 genes in this region, we identified 12 that showed differential expression between Z43 and Innovator tuber skins (Fig. 5A; Table S8), six of which overlapped with genes in cluster eight (Fig. 5B). Among these six genes, *Soltu.DM.04G003340* is homologous to *Arabidopsis PXG4*, which encodes a peroxygenase involved in lignin and suberin biosynthesis [32]. To explore the potential role of *StPXG4* in the regulation of tuber skin texture, we examined its expression levels in tuber skins of commercial varieties (Fig. 5C), including three smooth-skinned (Z43, Zhongshu 331, and Favorita), three weak russet-skinned (Atlantic, Zhongshu 28, and Zhongshu 49), and three russet-skinned varieties (Blazer Russet, Innovator, and Ranger Russet). Interestingly, *StPXG4* expression was significantly higher in smooth-skinned varieties than in russet-skinned ones (Fig. 5D), suggesting that this gene may play a universal role in regulating potato skin texture.

Notably, a total of 50 TFs were identified in cluster eight (Table S9). Based on Pearson correlation coefficients (>0.85) and *P*-values (<0.05), we constructed a correlation network linking *StPXG4* with these TFs (Fig. 5E). In total, 31 TFs showed strong correlations with *StPXG4*, among which two MYB TFs (*StMYB103* and *StMYB58*) were identified. The *Arabidopsis* homologs of *StMYB103* and *StMYB58* have previously been reported to regulate lignin biosynthesis [33, 34]. Interestingly, we identified three MYB binding sites in the promoter region of *StPXG4* (Table S10). These findings suggest that the expression of *StPXG4* may be regulated by *StMYB103* and/or *StMYB58*, thereby influencing tuber skin texture. However, further studies are needed to elucidate their precise biological roles in this regulatory process. Overall, *StPXG4* is a potential candidate gene for modulating tuber skin texture in potato.

## Discussion

Potato skin, the outermost protective layer of the tuber, plays a crucial role in resistance to both biotic and abiotic stresses. It is also an important trait influencing tuber appearance quality. Understanding the genetic basis and identifying functional genes underlying potato skin texture are essential for quality breeding and targeted improvement. In this study, we identified tuber skin texture QTL on Chromosome 4, where four indel markers linked to russeting were developed. Among these, three markers exhibited a concordance rate of up to 90% in pedigrees, providing valuable tools for marker-assisted selection in potato improvement programs. Furthermore, we demonstrated that suberin and lignin contents in potato tuber skin, as well as the structure and organization of the skin layers, are key factors influencing skin texture. Notably, *StPXG4* was identified as a critical regulator, with *StMYB103* and *StMYB58* emerging as potential upstream regulators of *StPXG4*. Collectively, our findings provide new insights into the molecular mechanisms controlling potato skin texture and can promote marker-assisted breeding for potato appearance quality.

### The cracking of potato tuber skin without shedding is a major contributing factor to russeting

During the early stages of tuber development, the outermost layer is the epidermis [35]. As the tuber grows to ~1–2 mm in diameter, the periderm gradually replaces the epidermis [36]. The periderm serves as a secondary protective tissue in certain plant species and organs. The potato tuber periderm serves as a widely used model system for studying phellem development, facilitated by the relative ease of isolating the skin (phellem) layer from the underlying periderm tissues [37], with significant variations in periderm structure observed among tuber skins with different textures. In most varieties, the number of phellem layers remains relatively stable during tuber development [38]. However, in cultivars such as ‘Russet Burbank’, ‘May Queen’, and ‘Winston’, the cracked phellem does not detach but instead adheres to the newly formed phellem layers, resulting in an increased number of phellem layers [9, 19, 39] and a consequent increase in tuber skin roughness [40].

In this study, we observed that the number of cell layers in the tuber skin of Innovator gradually increased as the tuber expanded. Concomitantly, during tuber expansion, the tuber skin of Innovator progressively developed cracks. The tissue adjacent to these cracks curled upward while remaining attached to the underlying, newly formed phellem layer. Although new phellem layers are continuously generated during tuber development [41], the number of phellem cell layers in Z43 remained stable throughout the developmental stages, with no obvious senescent or detaching layers observed. This phenomenon may be attributed to the efficient shedding of older periderm cells in Z43, which might be easily removed during sampling and subsequent washing steps. Collectively, these findings establish the persistent adherence and failed sloughing of older periderm layers as a central mechanistic determinant of russet skin architecture.

### The levels of suberin and lignin are closely associated with the tuber skin texture

It has been reported that cell wall components play critical roles in processes such as cell abscission, cell arrangement, and related developmental events [42]. The expression levels of cell wall-related genes between smooth- and russet-skinned varieties are significantly different [28, 43, 44]. Notably, when the russet tuber skin becomes smooth, genes involved in suberin biosynthesis are markedly upregulated [28]. In our study, we found that the DEGs in Innovator and Z43 during tuber development were significantly enriched in the lignin and fatty acid metabolic pathways. Given that lignin is a major structural component of the cell wall and fatty acids serve as key precursors for suberin synthesis [45, 46], these findings indicate that the biosynthesis of cell wall constituents may be a pivotal factor influencing the texture of potato tuber skin.

Suberin is an important component of tuber skin, accounting for 38% of the total tuber skin mass [17]. It is composed of two distinct polymeric domains: the poly(phenolic) domain (SPPD) and poly(aliphatic) domain (SPAD) [47]. The SPPD originates from the phenylpropanoid pathway and consists primarily of hydroxycinnamic acids and their derivatives, especially ferulic acid, as well as monolignols [48]. The SPAD, derived from the fatty acid pathway, mainly consists of very-long-chain fatty acids (VLCFAs) and their derivatives, including ω-hydroxyacids, dicarboxylic acids, primary alcohols, and glycerol [49]. Although the relative abundance and composition of suberin vary significantly across plant species and tissues, the core monomeric building blocks remain largely conserved [50].

In this study, we quantified eight major suberin monomers and found that the levels of fatty acid-derived monomers, specifically 18-hydroxy-9-octadecenoic acid and octadec-9-enedioic acid, were significantly lower in Innovator compared to Z43. Additionally, ferulic acid content was also reduced in Innovator. These results closely resemble the suberin profile observed in *FHT* gene interference (*FHT-RNAi*) potatoes [23]. These findings indicate that both the aromatic and aliphatic pathways of suberin formation are differentially regulated in smooth- and russet-skinned tubers.

Cellulose, hemicellulose, lignin, and pectin are fundamental structural elements of the cell wall [51]. Studies on apples have demonstrated that russet fruit skins exhibit higher levels of lignin and hemicellulose compared with smooth skin ones, along with an increased activity of enzymes associated with pectin and cellulose metabolism [42, 52]. In our analysis of these four components in potato tuber skins, only acid-soluble lignin and hemicellulose showed significant differences between Innovator and Z43. Notably, lignin content was substantially higher in Innovator. Interestingly, previous studies have reported a correlation between suberin and lignin accumulation, where changes in suberin deposition were often accompanied by parallel shifts in lignin levels [27, 53]. The differences in lignin and suberin contents between Innovator and Z43 tuber skins were consistently observed in the skins of multiple extreme offspring. Collectively, these findings indicate that suberin and lignin are the two most influential components in determining tuber skin texture in potatoes.

### *StPXG4* is a key candidate gene regulating tuber skin texture

Research on potato tuber skin texture dates back to the early 19th century [12], yet the elucidation of its genetic basis remains limited. Although genetic intervals associated with tuber skin texture have been mapped to nine chromosomes by Pandey, Vexler, and colleagues [14–16], the key regulatory loci underlying this trait remain to be conclusively identified. The three-gene interaction model proposed by De Jong and Pavek suggests that potato tuber skin texture is governed by three independently segregating dominant loci and that some smooth-skinned parental lines may carry one or two of these russet-associated alleles [13, 54]. Under this genetic model, mutation at any single locus among these three loci is sufficient to confer the russet phenotype. Accordingly, we hypothesized that a major genetic interval may play a predominant role in determining tuber skin texture. In this study, we developed an F₁ population using Innovator and Z43 and identified a major QTL on Chromosome 4 (1.86–3.80 Mb). Furthermore, the reliability of this identified locus was validated through molecular marker analysis.

To identify candidate genes within the QTL, we integrated QTL-gene mapping with transcriptome profiling of tuber skins from Innovator and Z43 across developmental stages, along with functional annotation of homologous genes. This integrative approach identified *StPXG4* as the key candidate gene. *StPXG4* is homologous to a caleosin-type peroxygenase gene in *Arabidopsis* that catalyzes the epoxidation of unsaturated fatty acids to produce epoxy fatty acids, which are key precursors in suberin biosynthesis [32, 55]. Notably, *StPXG4* expression was significantly higher in Z43 than in Innovator. This trend was consistently observed across seven additional commercial varieties: smooth-skinned varieties exhibited markedly higher *StPXG4* expression compared to russet-skinned varieties, suggesting a conserved role for *StPXG4* in determining skin texture in potatoes.

Furthermore, co-expression network analysis based on transcriptomic data revealed two MYB TFs, *StMYB103* and *StMYB58*, whose expression patterns were strongly correlated with *StPXG4*. Members of the MYB family are well-established regulators of suberin and lignin biosynthesis [34, 56]. Recent studies have demonstrated that *StMYB24*, *StMYB144*, and *StMYB168* positively regulate suberin deposition at wound sites during tuber healing [26]. Additionally, the *Arabidopsis* homolog of *StMYB103* is known to promote lignin biosynthesis [33]. Importantly, three MYB binding sites were identified in the promoter region of *StPXG4*, supporting the hypothesis that *StMYB103* and *StMYB58* may directly regulate its expression. Nevertheless, further research is required to confirm these regulatory interactions.

Collectively, our study identified a potato tuber skin texture QTL, revealed *StPXG4* as a key candidate gene, and proposed a potential regulatory module involving *StMYB103* and *StMYB58*. Nevertheless, further interesting questions are raised: What is the precise molecular function of *StPXG4* in potato? Is its role conserved across plant species? And what other regulatory factors, beyond the identified MYBs, contribute to the modulation of suberin or lignin biosynthesis? Addressing these questions will deepen our understanding of the genetic architecture underlying tuber skin texture and support breeding programs aimed at improving potato skin quality.

## Materials and methods

### Plant materials and growth conditions

The parents (Innovator and Z43) and their F_1_ population, consisting of 272 individuals, were planted under complete randomized block design in Fengning Manzhu Autonomous County, Hebei Province, China, and Zhenglan Banner, Inner Mongolia Autonomous Region, China, over two consecutive years (2023 and 2024). Each genotype was with three replicates. Each replicate consisted of a single row of four plants per genotype. The commercial varieties were grown in greenhouses under a controlled photoperiod of 16 h of light/8 h of dark at a constant temperature of 20°C in complete randomized block with three replicates. Each replicate consisted of three plants per variety.

### Phenotype analysis

At physiological maturity, skin texture of all F_1_ individuals was visually scored on a 1–5 scale (1: smooth; 2: slightly smooth; 3: medium; 4: slightly russet; 5: russet) following the National Standard for DUS Testing of Potato Varieties (GB/T 19557.28). This morphological assessment was performed solely by the first author to ensure scoring consistency. To ensure phenotypic consistency, only individuals that were repeatedly scored as 1–2 or 4–5 across environments were considered as extreme smooth or extreme russet, respectively, and were subsequently used for bulk construction and phenotype prediction accuracy analysis.

### Microscopic observation

For SEM, samples were fixed in glutaraldehyde at room temperature (22 ± 1°C) for 48 h. The specimens were then dehydrated in a graded ethanol series, exchanged through amyl acetate, and subjected to critical point drying. After gold sputter coating, samples were observed at 15 kV using a TM4000 scanning electron microscope (Hitachi High-Technologies Corp., Tokyo, Japan).

For light microscopy, samples were fixed overnight in 3.7% formalin–acetic acid–alcohol, dehydrated in an ethanol series, cleared in xylene, and embedded in paraffin. Longitudinal skin sections were cut, stained with Safranin-O/Fast green (Servicebio, Wuhan, China), and observed under bright-field illumination using a BX51 optical microscope (Olympus Corp., Tokyo, Japan).

### Suberin monomer analyses

Suberin content was determined following established protocols [57]. Briefly, 5–10 mg of dry sample powder was suspended in 1 ml of dichloromethane–methanol (1:1, v/v) solution and incubated at 50 rpm for 16–18 h to extract waxes. After solvent removal, the wax-free solid residue was subjected to methanolic boron trifluoride (BF_3_/MeOH) and incubated at 70°C for 18 h to depolymerize the suberin via transesterification. Subsequently, 10 μg of dotriacontane, used as an internal standard, was added, and the resulting mixture was transferred to 2 ml of saturated aqueous sodium hydrogen carbonate (NaHCO_3_) solution. The residual pellet was then rinsed with dichloromethane, and the rinses were combined with the mixture. After phase separation, the organic layer was collected, and the solvent was evaporated to dryness under nitrogen or vacuum. The dried residue was then derivatized by adding 50 μl each of N-methyl-N-(tert-butyldimethylsilyl) trifluoroacetamide (MTBSTFA) and pyridine, followed by incubation at 120°C for 60 min. Compounds were identified using an Agilent 7890B gas chromatograph coupled to an Agilent 7000A triple quadrupole mass spectrometer (Agilent Technologies, Santa Clara, CA, USA), and data were analyzed with Agilent MassHunter Qualitative Analysis (v10.0) and Agilent MassHunter Unknowns Analysis (v10.1). Suberin monomers were identified based on the NIST17 mass spectral library (National Institute of Standards and Technology, Gaithersburg, MD, USA). The concentration of individual suberin monomers was quantified by comparing their chromatographic peak areas to that of the internal standard. Final monomer contents were normalized to the dry weight of the original tissue samples. Each biological replicate consisted of tuber skin collected from at least three distinct tubers, and three biological replicates were analyzed per individual.

### Cell wall composition analyses

Cell wall components were extracted and analyzed as described previously [58]. Dry powdered skin samples were subjected to ethanol extraction using the Soxhlet method for 12 h and then dried at 105°C. To 100 mg of powder, 1.5 ml of 72% sulfuric acid was added, followed by shaking for 30 min. Subsequently, 42 ml deionized water was added, and the mixture was heated at 121°C for 30 min. Lignin content was determined by filtering the reaction mixture, drying the residual solid at 105°C, and measuring its weight. The supernatant was used to quantify galacturonic acid, glucose, arabinose, and xylose, which were used to calculate the contents of pectin, cellulose, and hemicellulose, respectively.

### Bulked segregant analysis

Twenty-nine individuals with russet skin and thirty individuals with smooth skin were selected from the F_1_ population. Genomic DNA was extracted from each individual and equimolarly pooled to construct the russet and smooth DNA pools. Whole-genome resequencing of parents and extreme pools was performed on the Illumina NovaSeq 6000 sequencing platform (Illumina Inc., San Diego, CA, USA). Raw sequencing reads were quality-trimmed and filtered using Fastp (v0.23.4) [59]. High-quality clean reads were then aligned to the DM6.1 reference potato genome (https://spuddb.uga.edu/data/dm_v61/DM_1-3_516_R44_potato_genome_assembly.v6.1.fa.gz) using BWA (v0.7.18) [60]. Duplicate reads were marked and removed, and variant calling was performed using the Genome Analysis Toolkit (GATK v4.5) [61]. The resulting VCF files were analyzed and visualized with DeepBSA (v1.0) and OcBSA (v1.4) to identify the QTL [62, 63]. Variants were functionally annotated using SnpEff (v5.2e) [64]. *k*-mer analysis was performed using the CoSSA (v4.0) software with default parameters [65]. Indel markers were developed based on polymorphisms identified between the parental and pool sequences. Candidate markers linked to the tuber skin trait were validated for polymorphism using DNA from the primary segregating population and parental lines.

### Transcriptome data analysis

Tuber skin samples from three developmental stages were collected for RNA-Seq analysis. Each sample included two biological replicates, with each replicate comprising skin tissue pooled from five individual tubers. Total RNA was extracted using TRIzol reagent (Invitrogen, Carlsbad, CA, USA), and RNA quality was evaluated using the NanoDrop® 2000 spectrophotometer (Thermo Fisher Scientific, Wilmington, DE, USA), while integrity was verified by 0.8% agarose gel electrophoresis. High-quality RNA samples were used to construct cDNA libraries, which were then sequenced on the Illumina NovaSeq 6000 platform (Illumina Inc., San Diego, CA, USA). Raw sequencing reads were processed to obtain clean data by removing adapters and low-quality reads using Fastp (v0.23.2). Clean reads were aligned to the DM v6.1 reference genome using HISAT2 (v2.2.1) [66]. Gene expression levels were quantified as fragments per kilobase of transcript per million mapped reads (FPKM) using FeatureCounts (v2.0.3) [67]. DEGs were identified with a threshold of false discovery rate (FDR) <0.05 and |log2 fold change| ≥ 2. All DEGs were clustered into distinct expression patterns based on their FPKM values using the stats package (v4.2.0) in R. Functional enrichment analyses, including GO and KEGG pathway annotations, were conducted using ClusterProfiler (v4.6.0) [68].

### Correlation network analysis

The network displayed only nodes with Pearson’s correlation coefficient (*r*) >0.85 and *P*-values <0.05 in relation to *StPXG4*. The final network was visualized using Cytoscape (v3.9.1) [69].

### Quantitative real-time PCR analysis

Total RNA was extracted from samples ground in liquid nitrogen using TRIzol reagent. The extracted RNA was then reverse-transcribed into cDNA using HiScript reverse transcriptase (Vazyme Biotech Co., Ltd, Nanjing, China). For each sample, at least three technical and three biological replicates were included in the analysis. The primer sequences used for qRT-PCR are listed in Table S11.

### Language editing

The language of this manuscript was improved with the assistance of ChatGPT (GPT-5.2, OpenAI’s large-scale language model). All AI-assisted edits were carefully reviewed and revised by the authors, who assume full responsibility for the final content.

## Supplementary Material

Web_Material_uhag102

## Data Availability

The genomic sequence raw data are available from NCBI under BioProject accession No. PRJNA1416846.
