# Supplementary figures and images for: Genetic architecture and major genes for tuber skin texture in potato
Source: Hortic Res. 2026 Mar 13;13(7):uhag102. doi: 10.1093/hr/uhag102 (PMC13283846; doi:10.1093/hr/uhag102)

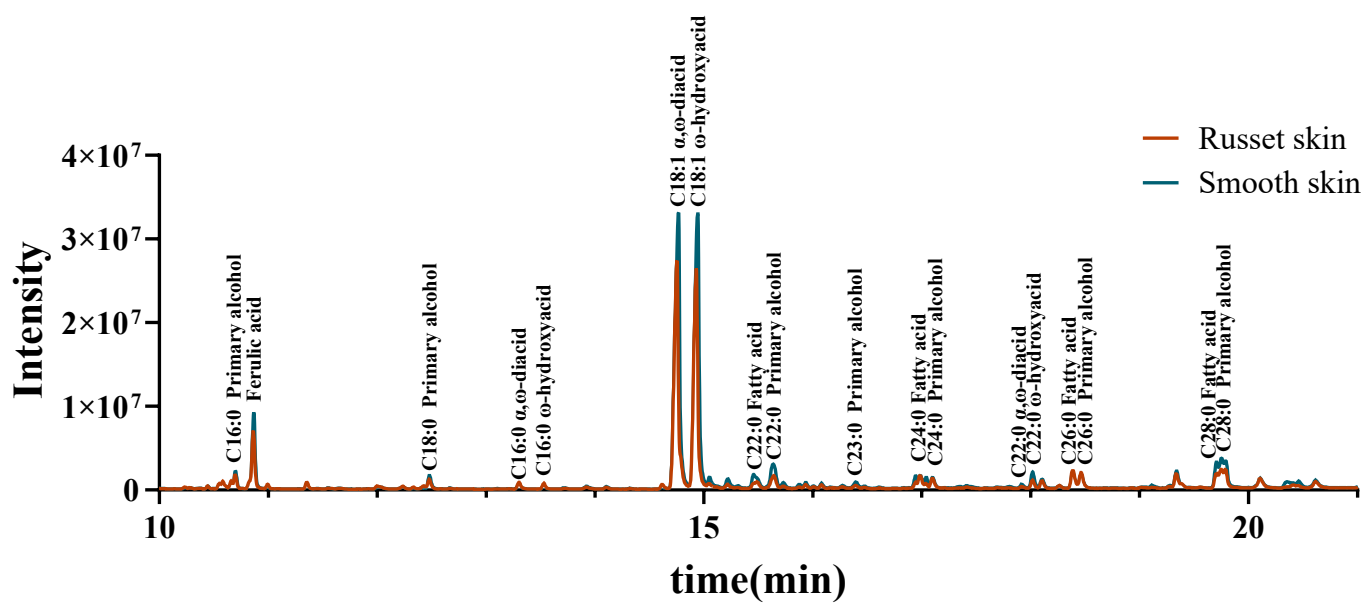

Supplement: Web_Material_uhag102 [file web_material_uhag102.zip › Supplementary Figure 1.pdf]

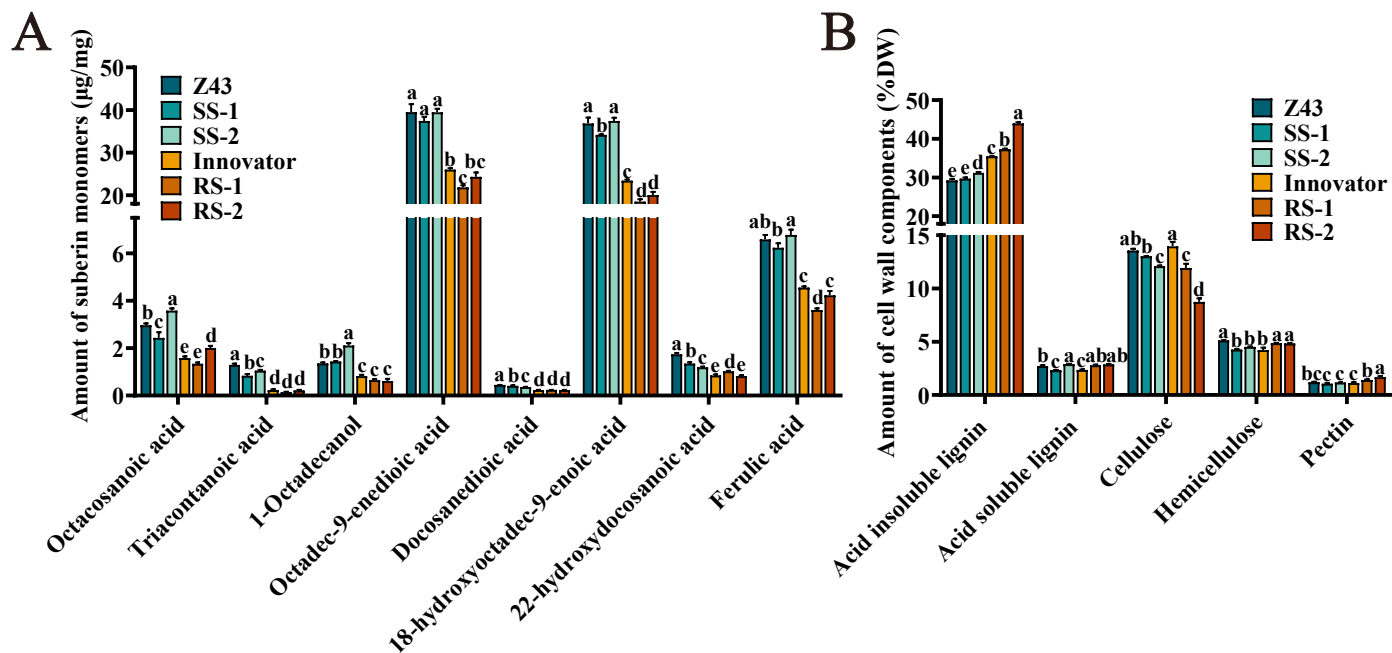

Supplement: Web_Material_uhag102 [file web_material_uhag102.zip › Supplementary Figure 2.pdf]

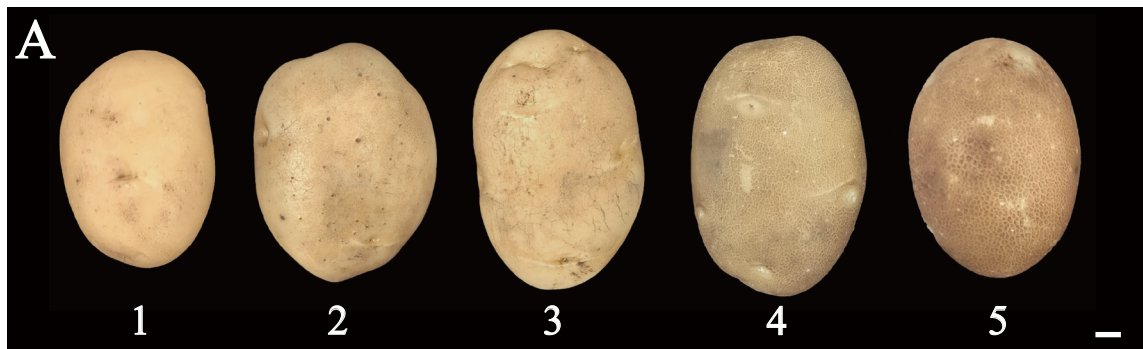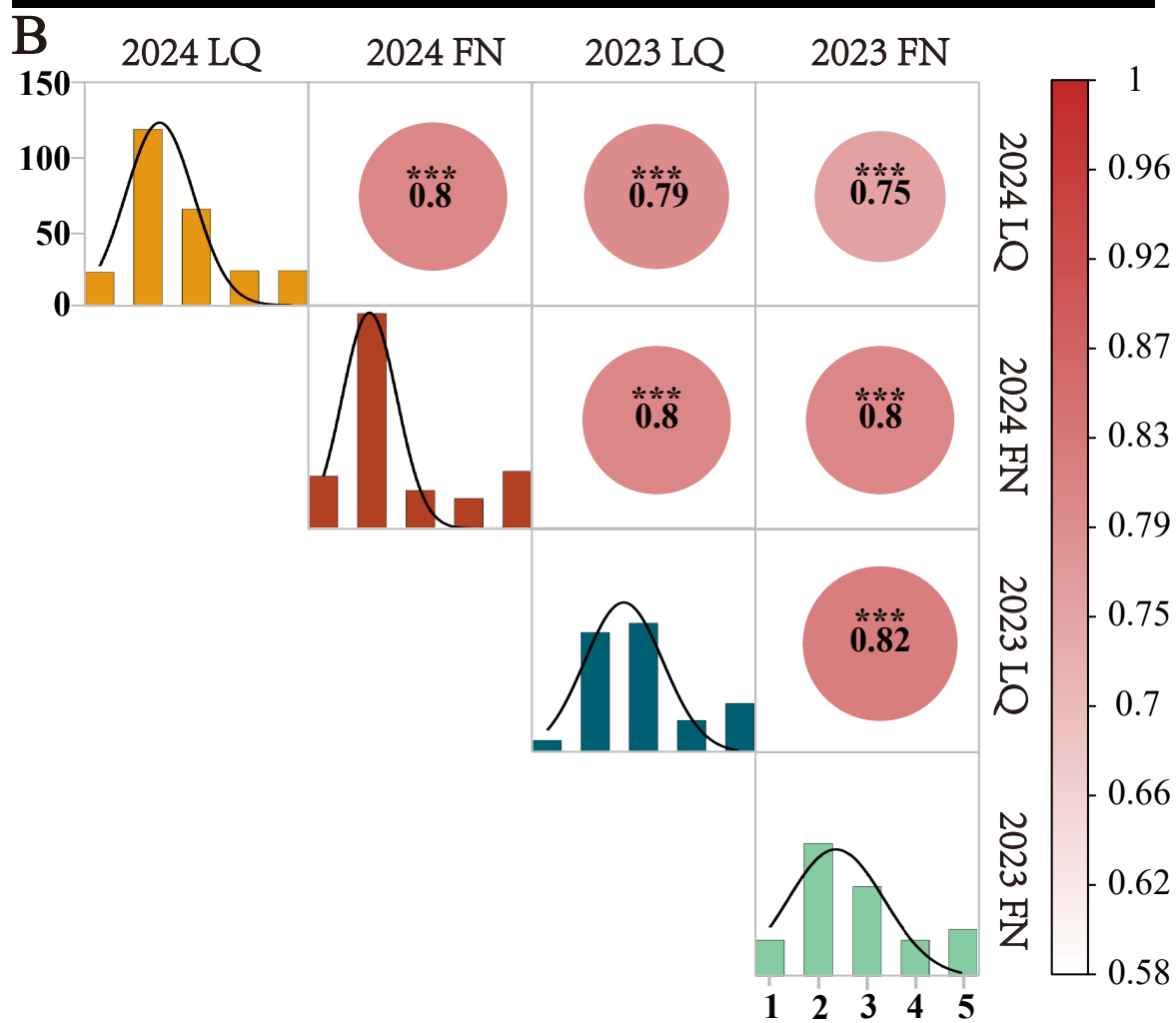

Supplement: Web_Material_uhag102 [file web_material_uhag102.zip › Supplementary Figure 3.pdf]

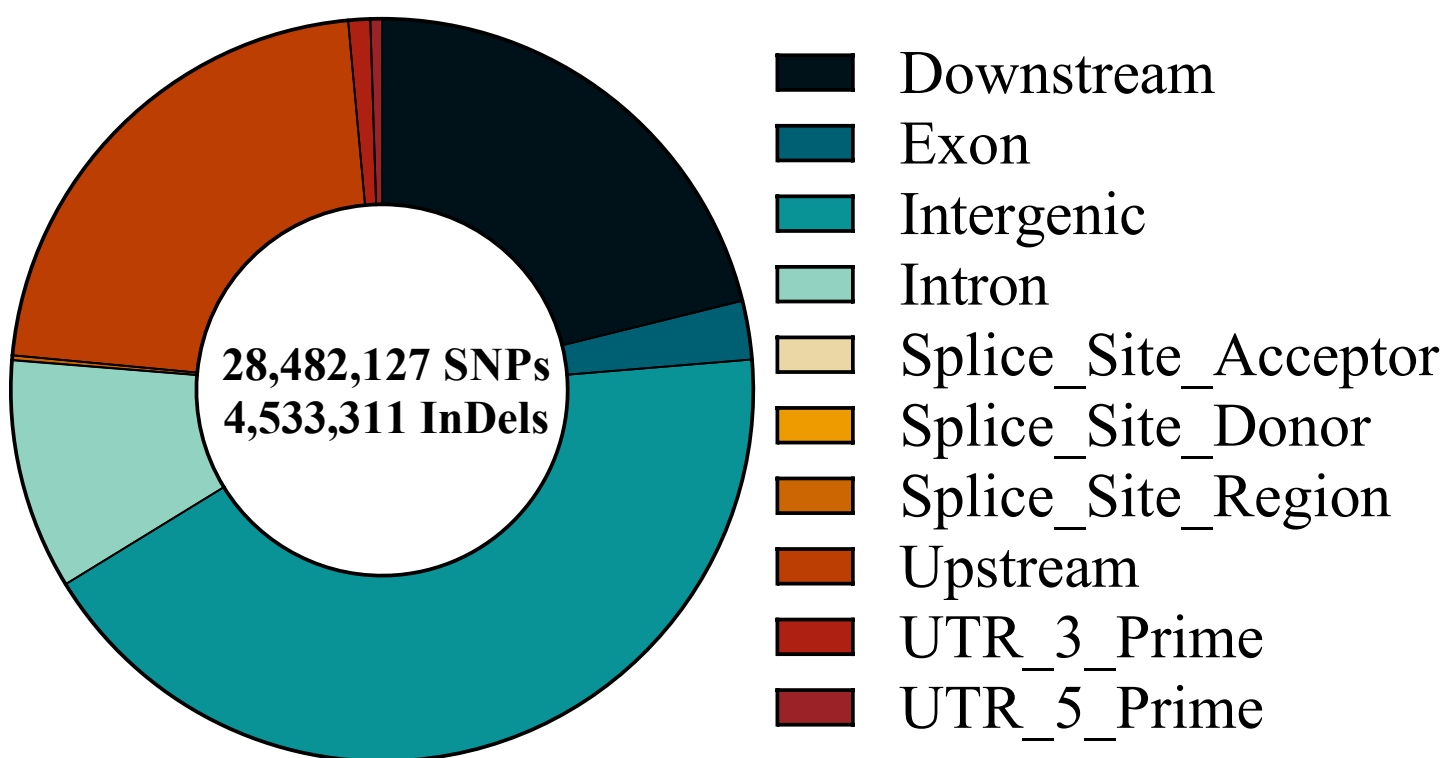

Supplement: Web_Material_uhag102 [file web_material_uhag102.zip › Supplementary Figure 4.pdf]

# The number of SNPs within 1Mb window size

0Mb 9.8Mb 19.6Mb 29.4Mb 39.2Mb 49Mb 58.8Mb 68.6Mb 78.4Mb 88.2Mb

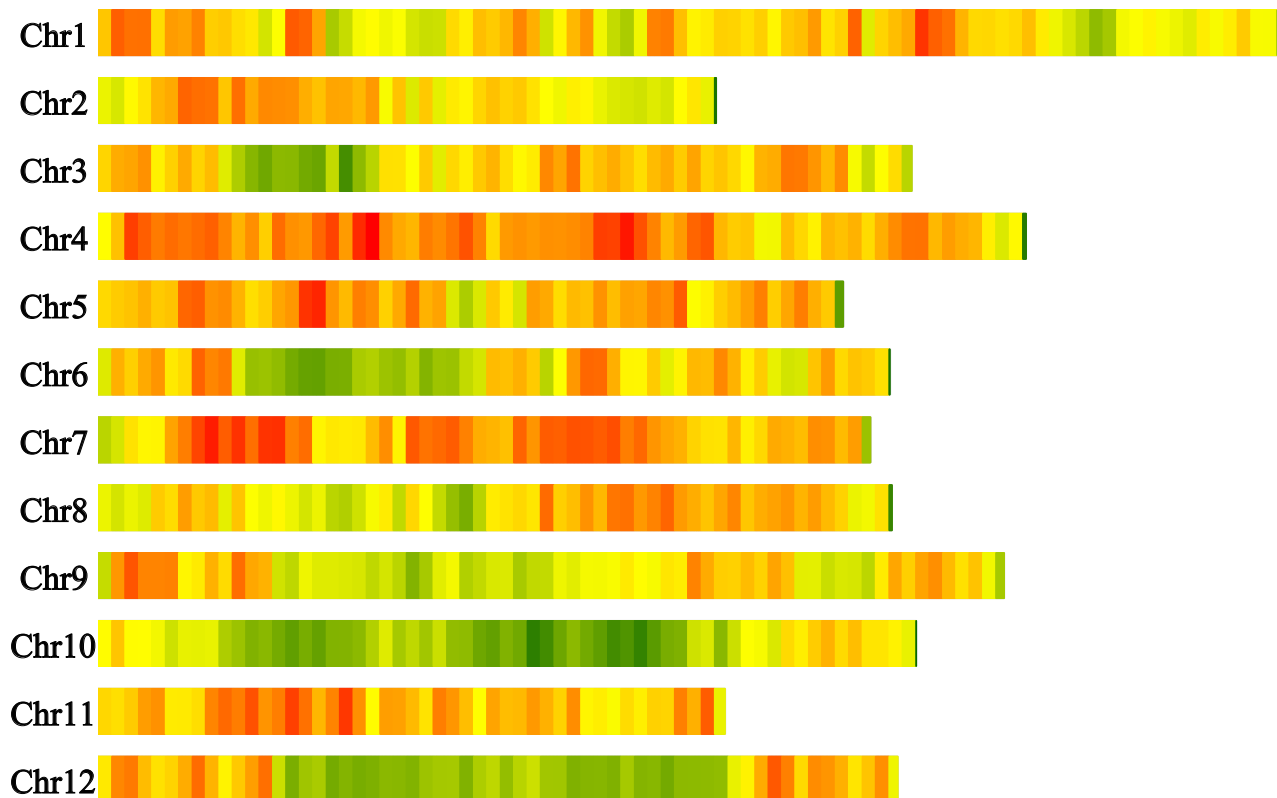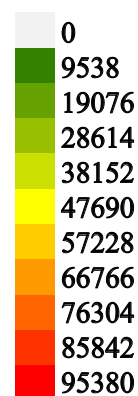

Supplement: Web_Material_uhag102 [file web_material_uhag102.zip › Supplementary Figure 5.pdf]

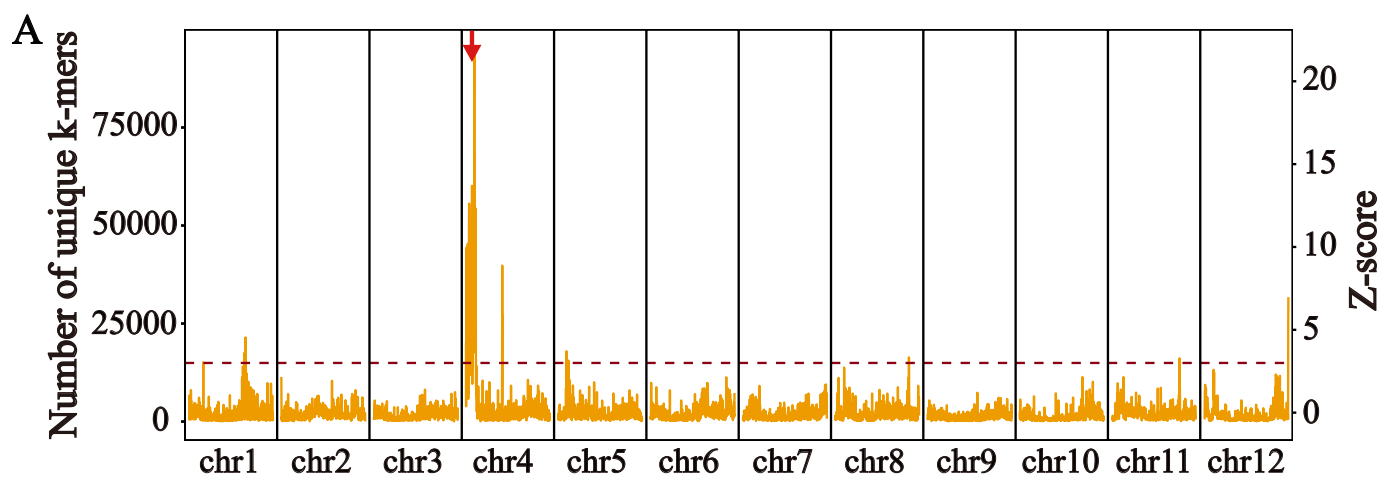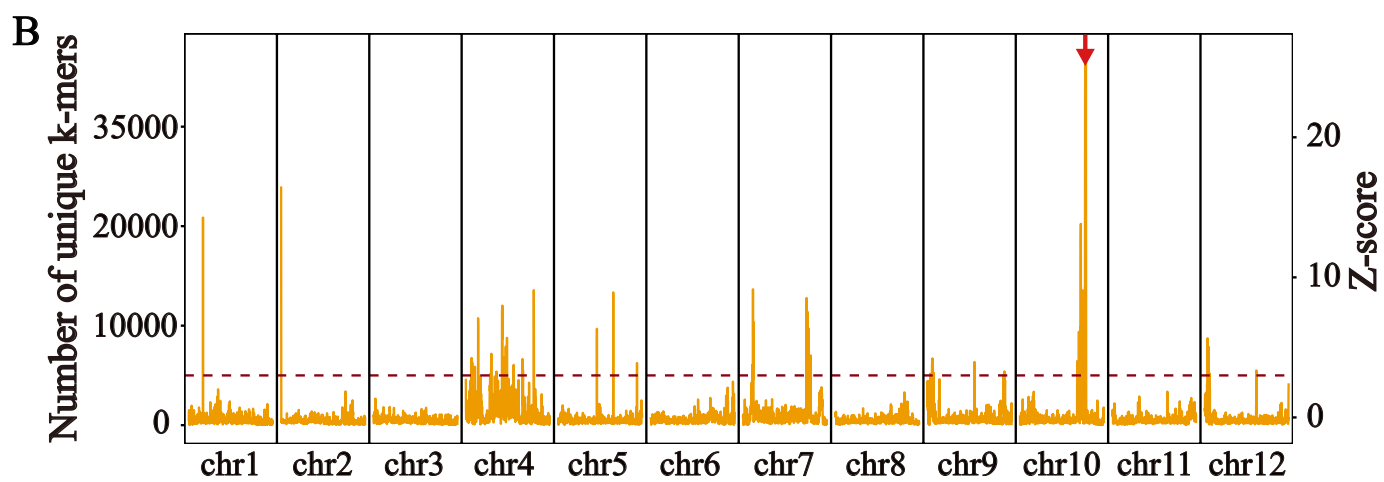

Supplement: Web_Material_uhag102 [file web_material_uhag102.zip › Supplementary Figure 6.pdf]

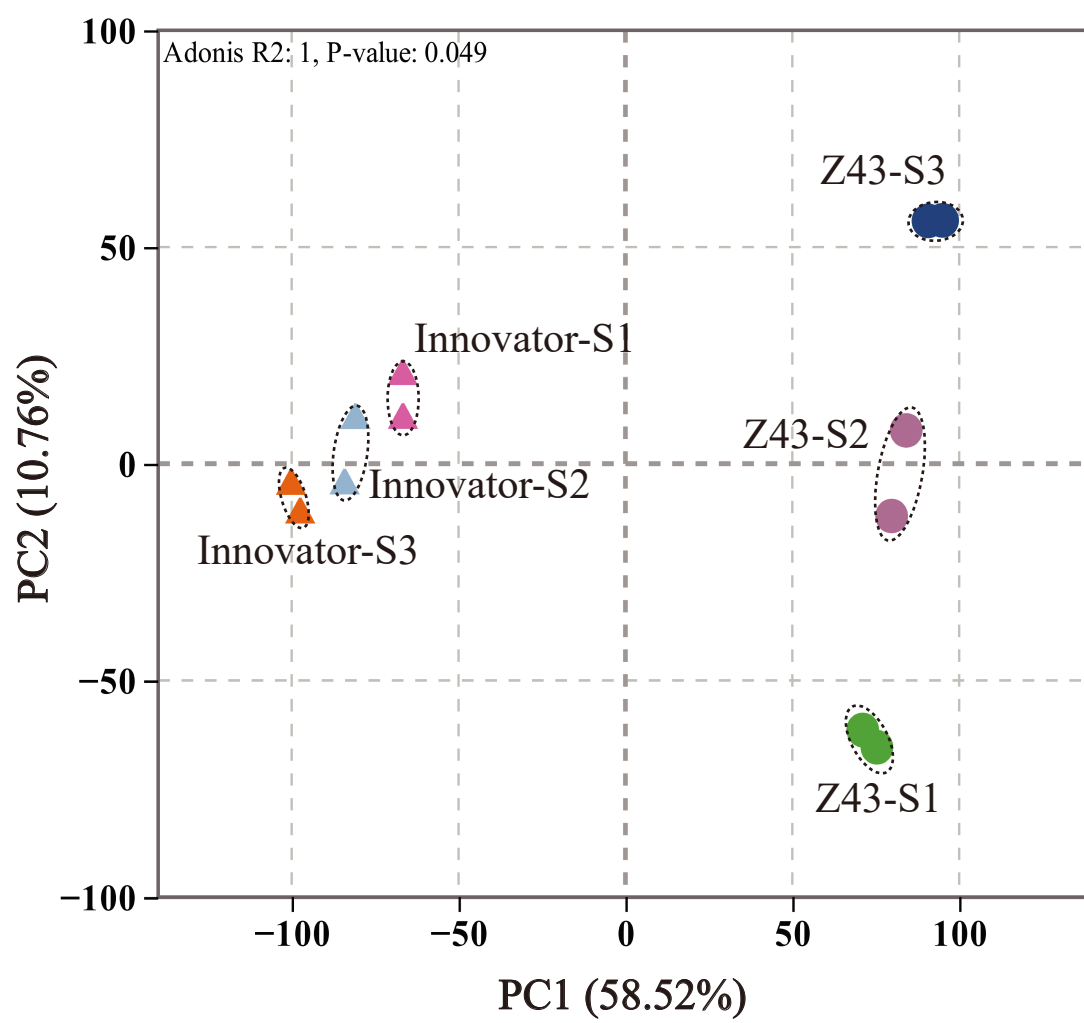

Supplement: Web_Material_uhag102 [file web_material_uhag102.zip › Supplementary Figure 7.pdf]
